# Supplementary figures and images for: iTRAQ-based quantitative proteome analysis reveals metabolic changes between a cleistogamous wheat mutant and its wild-type wheat counterpart
Source: PeerJ. 2019 Jun 17;7:e7104. doi: 10.7717/peerj.7104 (PMC6585907; doi:10.7717/peerj.7104)

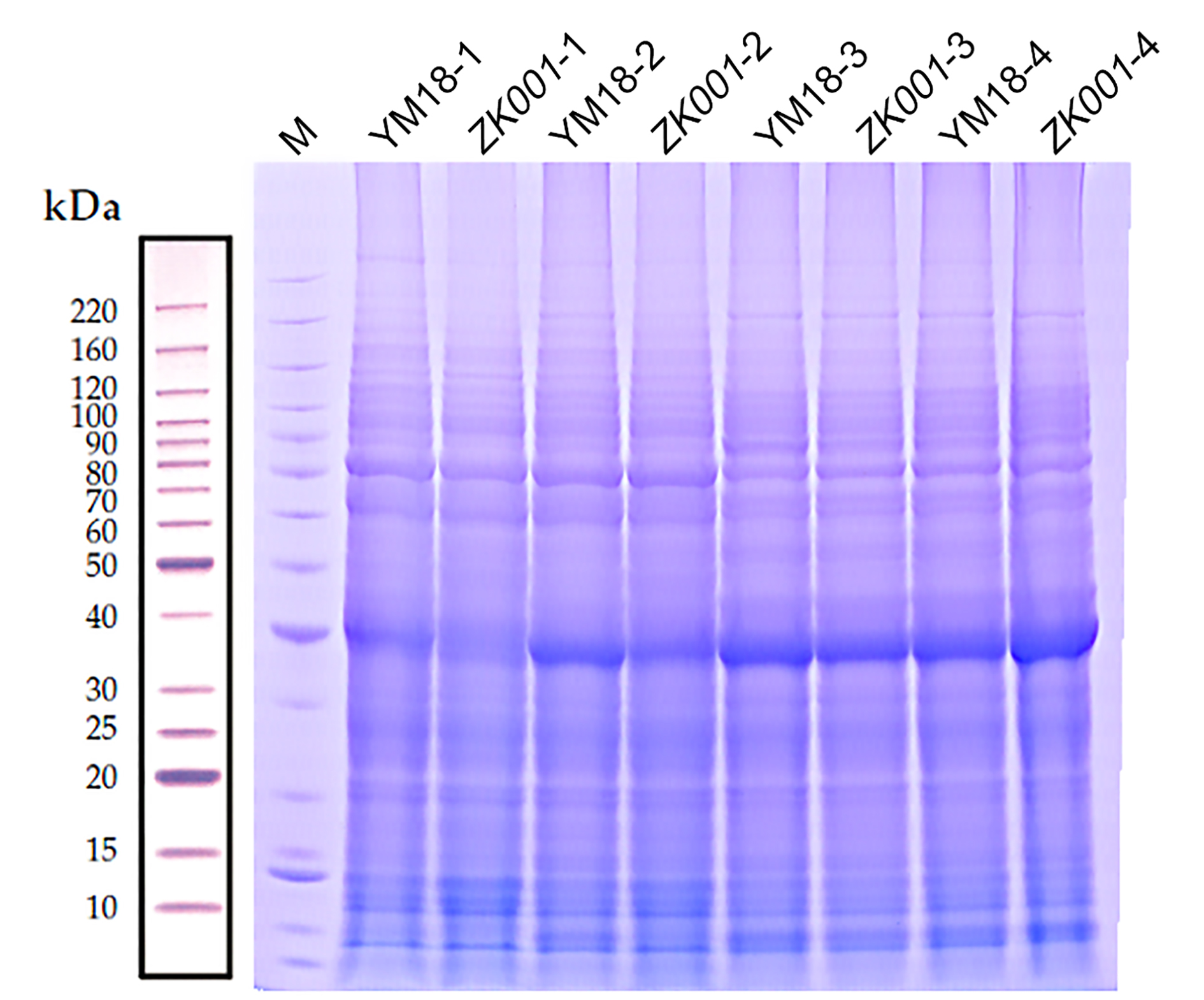

Supplement: Supplemental Information 1 — YM18-1: YM18-WAS; YM18-2: YM18-GAS; YM18-3: YM18-YAS; YM18-4: YM18-AS; ZK001-1: ZK001-WAS; ZK001-2: ZK001-GAS; ZK001-3: ZK001-YAS; ZK001-4: ZK001-AS. The total protein of 8 samples was effectively separated in the range of 10-220 kDa. Protein bands were clear, complete, uniform, and free degraded. Total protein can meet further experiments. [file peerj-07-7104-s001.jpg]

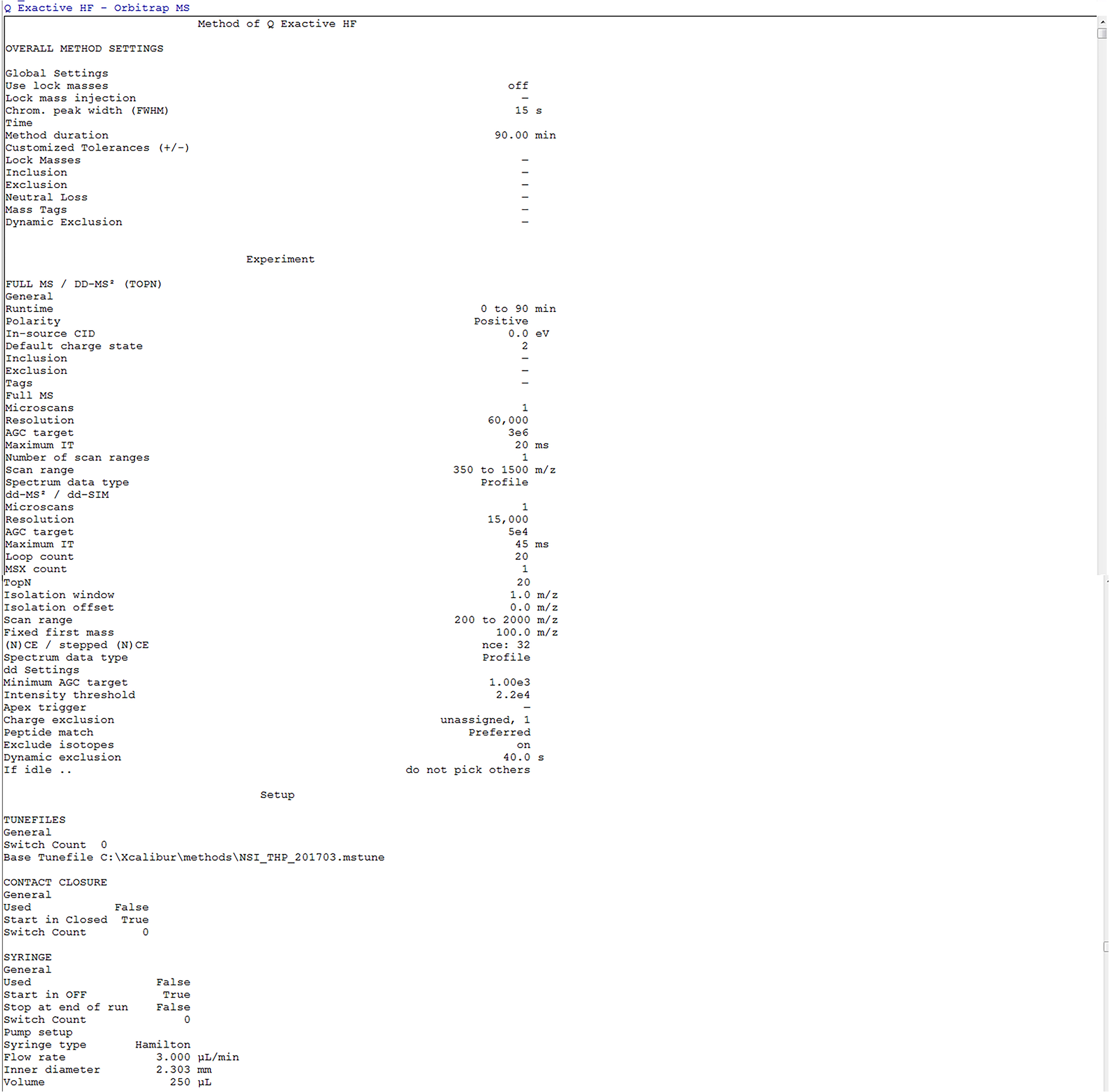

Supplement: Supplemental Information 2 — These parameters ensure the correct recognition of reporter ions with different isotopic labels. [file peerj-07-7104-s002.jpg]

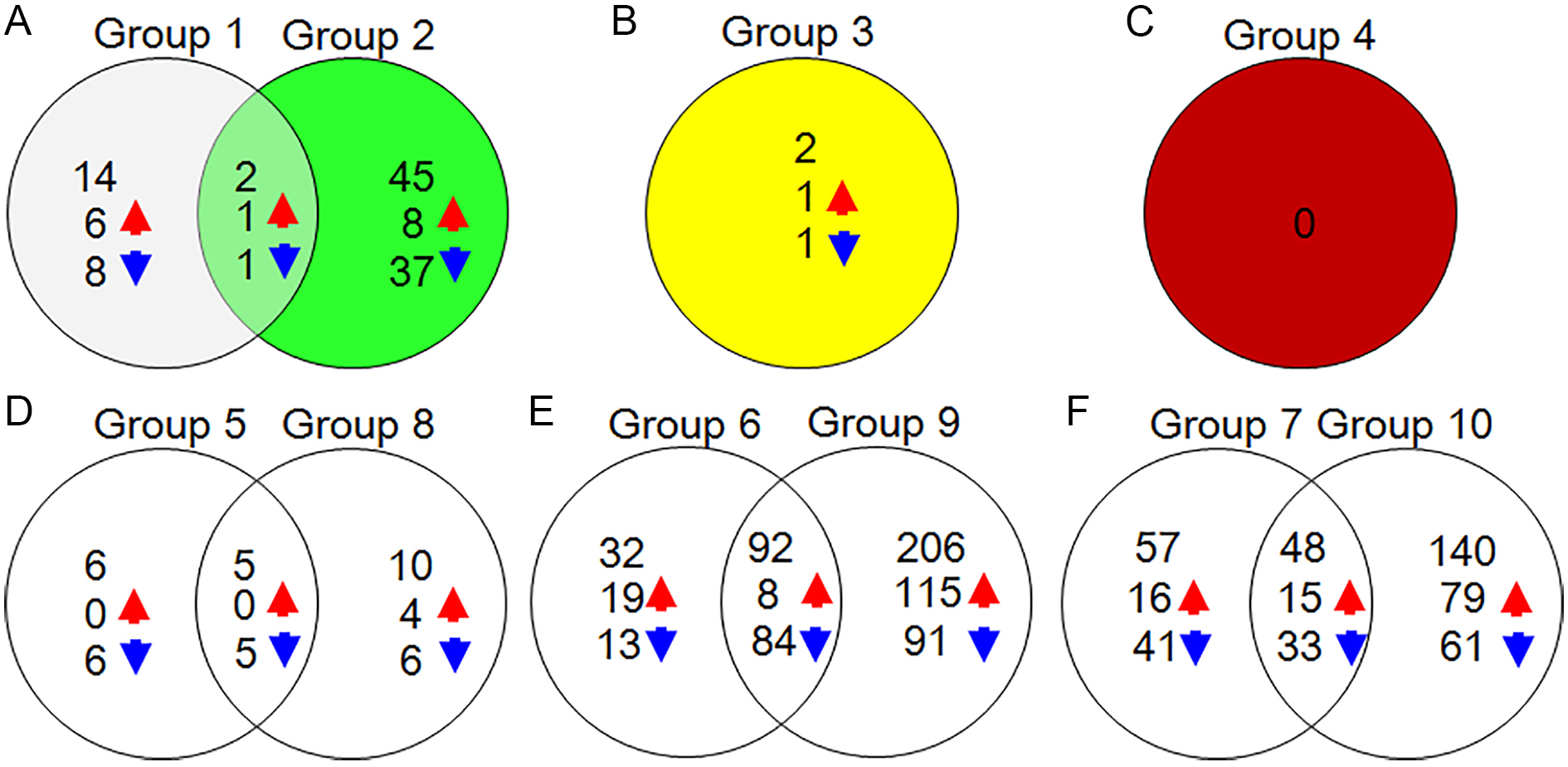

Supplement: Supplemental Information 3 — A total of 14 and 45 specific proteins were identified from Group 1 and Group 2, respectively; 2 common proteins belonged to Group 1 and Group 2 (A). Group 3 (B) and Group 4 (C) showed no overlap with Group 1 or Group 2. Venn diagram of DAPs from Group 5 vs Group 8 (D), Group 5 vs Group 8 (E) and Group 5 vs Group 8 (F), respectively. The red and blue arrows represented increasing and decreasing -abundance proteins of ZK001 compared with YM18, respectively. (0.667 < FC < 1.5, corrected P–value < 0.01). [file peerj-07-7104-s003.jpg]

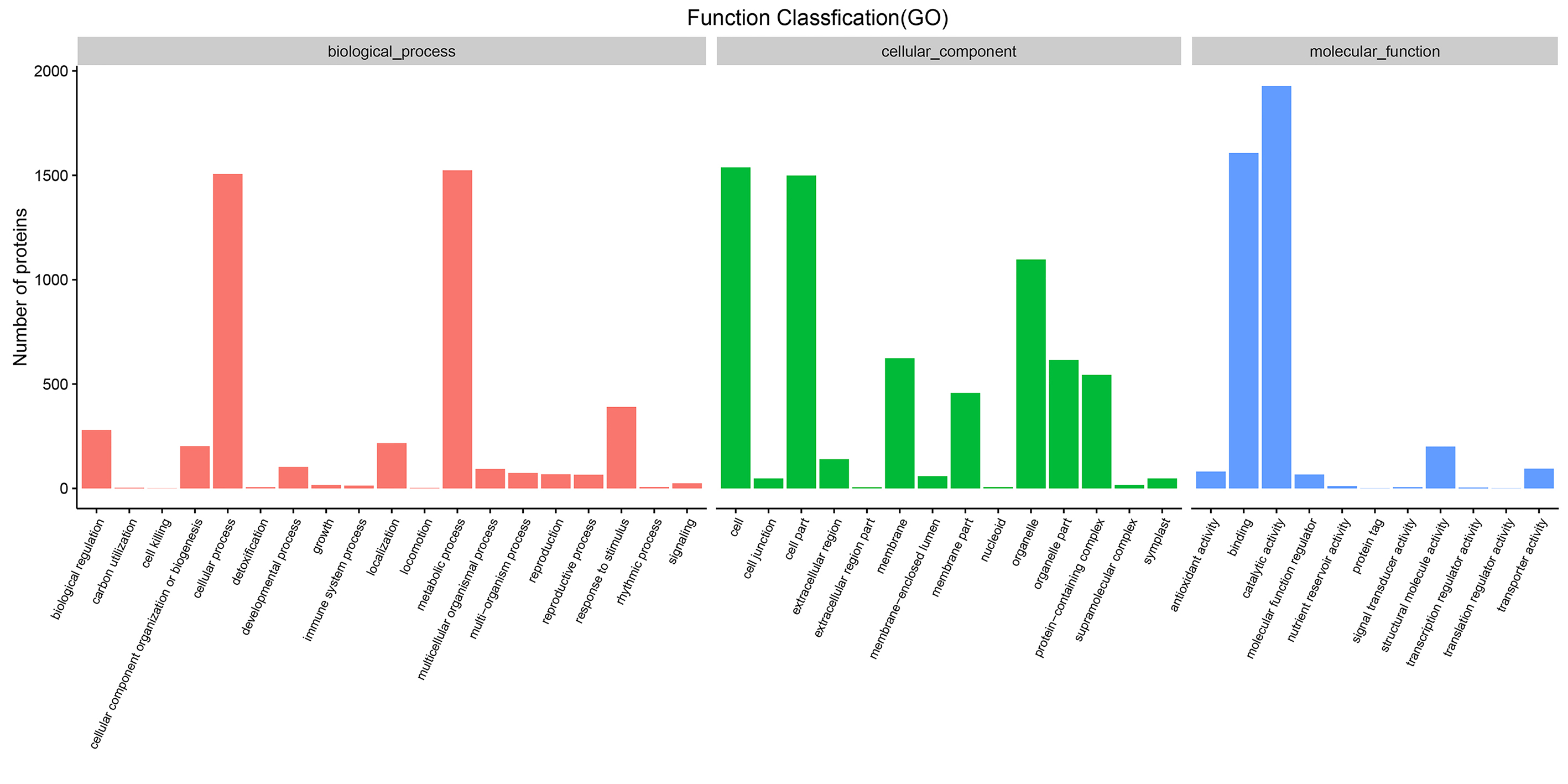

Supplement: Supplemental Information 4 — This figure shows that all of the identified proteins in YM18 and ZK001 are involved in 19 subgroups of biological process, 14 subgroups of cellular component, and 11 subgroups of molecular function. [file peerj-07-7104-s004.jpg]

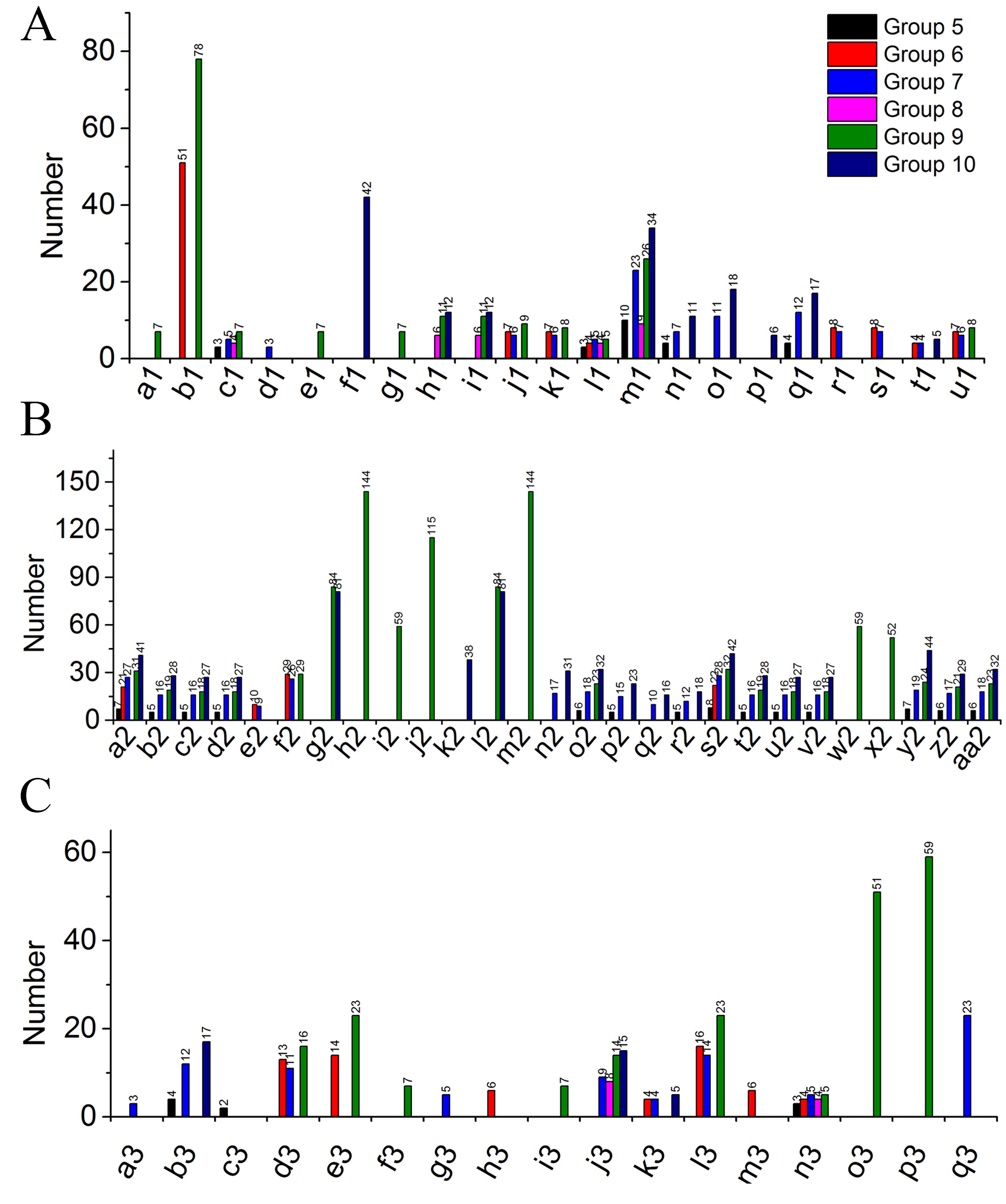

Supplement: Supplemental Information 5 — (0.667 < FC < 1.5, corrected P–value < 0.01) A: biological process of Group 5-10. a1: carbohydrate homeostasis; b1: carbohydrate metabolic process; c1: carbon fixation; d1: carbon utilization; e1: cellular glucose homeostasis; f1: generation of precursor metabolites and energy; g1: glucose homeostasis; h1: lipid localization; i1: lipid transport; j1: multi-organism process; k1: multi-organism reproductive process; l1: photorespiration; m1: photosynthesis; n1: photosynthesis, light harvesting; o1: photosynthesis, light reaction; p1: photosynthetic electron transport chain; q1: protein-chromophore linkage; r1: reproduction; s1: reproductive process; t1: S-adenosylmethionine biosynthetic process; u1: sexual reproduction; B: cellular component of Group 5-10. a2: chloroplast; b2: chloroplast part; c2: chloroplast thylakoid; d2: chloroplast thylakoid membrane; e2: cytoskeleton; f2: extracellular region; g2: intracellular non-membrane-bounded organelle; h2: intracellular organelle; i2: intracellular ribonucleoprotein complex; j2: macromolecular complex; k2: membrane protein complex; l2: non-membrane-bounded organelle; m2: organelle; n2: organelle subcompartment; o2: photosynthetic membrane; p2: photosystem; q2: photosystem I; r2: photosystem II; s2: plastid; t2: plastid part; u2: plastid thylakoid; v2: plastid thylakoid membrane; w2: ribonucleoprotein complex; x2: ribosome; y2: thylakoid; z2: thylakoid membrane; aa2: thylakoid part; C: molecular function of Group 5-10. a3: carbonate dehydratase activity; b3: chlorophyll binding; c3: electron transporter, transferring electrons within cytochrome b6/f complex of photosystem II activity; d3: enzyme inhibitor activity; e3: enzyme regulator activity; f3: glucose binding; g3: glucosidase activity; h3: glycogen phosphorylase activity; i3: hexokinase activity; j3: lipid binding; k3: methionine adenosyltransferase activity; l3: molecular function regulator; m3: phosphorylase activity; n3: ribulose-bisphosphate carboxylase activity; [file peerj-07-7104-s005.jpg]
